# Effects of betaine on body composition, performance, and homocysteine thiolactone

**DOI:** 10.1186/1550-2783-10-39

**Published:** 2013-08-22

**Authors:** Jason M Cholewa, Monika Wyszczelska-Rokiel, Rafal Glowacki, Hieronim Jakubowski, Tracey Matthews, Richard Wood, Stuart AS Craig, Vincent Paolone

**Affiliations:** 1 Department of Kinesiology, Recreation, and Sport Studies, Coastal Carolina University, Conway, SC, USA; 2 Department of Environmental Chemistry, University of Lodz, Pomorska 163, 90-236, Lodz, Poland, USA; 3 Institute of Bioorganic Chemistry, Polish Academy of Sciences, Poznan, Department of Biochemistry and Biotechnology, Life Sciences University, Poznan, NJ, Poland; 4 UNDMJ-New Jersey Medical School, Newark, NJ, USA; 5 Department of Exercise Science and Sport Studies, Springfield College, Springfield, MA, USA; 6 DuPont Nutrition & Health, Tarrytown, NY, USA

**Keywords:** Hypertrophy, Strength, Power, Supplementation, Trimethylglycine

## Abstract

**Background:**

This study investigated the effects of long term betaine supplementation on body composition, performance, and homocysteine thiolactone (HCTL) in experienced strength trained men.

**Methods:**

Twenty-three subjects were matched for training experience (4.8 ± 2.3 years) and body fat percentage (BF%: 16.9 ± 8.0%), randomly assigned to either a placebo (PL; *n* = 12) or betaine group (BET; *n* = 11; 2.5 g/day), and completed a 6 week periodized training program consisting of 3 two-week micro-cycles. Bench press and back squat training volumes were recorded and changes in training volume were assessed at each micro-cycle. Fasting urine was collected at baseline (BL), weeks 2, 4 and 6, and assayed for HCTL. Subjects were tested prior to and following 6 weeks of treatment. Arm and thigh cross sectional area (CSA) was estimated via girth and skin fold measurements. Body density was estimated via skin fold calipers and used to estimate BF%, fat mass (FM), and lean body mass (LBM). Performance was assessed via vertical jump (VJ), bench press 1 RM (BP), and back squat 1 RM (BS).

**Results:**

Arm CSA increased significantly (*p* < .05) in BET but not PL. No differences existed between group and time for changes in thigh CSA. Back squat training volume increased significantly (*p* < .05) for both groups throughout training. Bench press training volume was significantly (*p* < .05) improved for BET compared to PL at microcycles one and three. Body composition (BF%, FM, LBM) improved significantly (*p* < .05) in BET but not PL. No differences were found in performance variables (BP, BS, VJ) between groups, except there was a trend (p = .07) for increased VJ power in BET versus PL. A significant interaction (*p* < .05) existed for HCTL, with increases from BL to week 2 in PL, but not BET. Additionally, HCTL remained elevated at week 4 in PL, but not BET.

**Conclusion:**

Six-weeks of betaine supplementation improved body composition, arm size, bench press work capacity, attenuated the rise in urinary HCTL, and tended to improve power (p = .07) but not strength.

## Background

Betaine (trimethylglycine) is an organic osmolyte found in many foods, including spinach, beets, and whole grains [1]. Administration of supplemental betaine for 10–15 days has enhanced performance in several studies but with varying results: Lee et al. [2] reported increased power output and force production, whereas others [3,4] reported improvements in muscular endurance but not power. On the other hand, Del Favero et al. [5] reported no improvements in power output, strength, or body composition with 10 days of betaine treatment; however, subjects were instructed to avoid training and supplementation was ceased 5 days prior to performance testing.

To the author’s knowledge, only two studies have examined the effects of betaine on body composition and hypertrophy in humans. Betaine did not improve body composition in obese, sedentary subjects on a 500 kcal/day caloric deficit following 12 weeks of supplementation [6]. Similarly, 10 days of betaine supplementation did not improve body composition in sedentary young male subjects [5]. Though research is limited in humans, chronic betaine supplementation has been shown to reduce adipose mass and increase muscle mass in animals [7-9]. Greater improvements in body composition with betaine supplementation were observed when pigs were given extra pen space to move and exercise [9], suggesting that betaine may exert the most influential effects on growth under conditions of metabolic or nutritional stress. Because the subjects in Schwab et al. [6] and Del Favero et al. [5] were instructed not to exercise, the absence of a metabolic stressor may have compromised the effects of betaine. Given the enhanced effects of movement in pigs and the ineffectiveness reported in sedentary, non-exercising humans, we hypothesize that the effects of betaine on body composition, strength and power may be most apparent when supplementation occurs over several weeks accompanied by a resistance training program.

By donating a methyl group to transmethylate Hcy back to methionine (Met), betaine increases Hcy metabolism and the availability of the universal methyl donor, *S*-adenosylmethionine (SAM) [10]. We hypothesize betaine supplementation may enhance protein synthesis and thus improve body composition by reducing Hcy and homocysteine thiolactone (HCTL). Hcy directly impairs insulin signaling by reducing insulin receptor stubstrate-1 (IRS-1) activation and thus inhibiting Akt-phosphorylation [11]. Moreover, excess dietary Met is metabolized to form Hcy and both high dietary Met consumption and the resultant increase in plasma Hcy contributes to elevated HCTL [12]. A short (10 min) HCTL treatment inhibits insulin signaling, including insulin-mediated mRNA expression and protein synthesis [13]. This suggests that HCTL is more effective than Hcy in promoting insulin resistance. Additionally, HCTL has been shown to modify protein lysine residues, which causes protein aggregation, and inactivates enzymes associated with protein synthesis [14].

Concentrations of plasma Hcy or HCTL levels in strength athletes have yet to be reported. Given that transmethylation capacity is dependent upon plasma folate and betaine [15] and because weight trainers regularly consume excess Met and inadequate folate and betaine [16], Hcy transmethylation may be impaired resulting in excess HCTL generation. Thus, by decreasing insulin receptor signaling [11], elevated HCTL in weight lifters may compromise body composition directly by inhibiting mRNA expression and protein synthesis.

In healthy adults the ingestion of 500 mg of betaine decreased fasting plasma Hcy and attenuated Hcy rise for 24 hr following a Met load [11], and betaine treatment lowers HCTL in patients with genetically compromised transmethylation capacities [12]; however, to date there are no published reports investigating the effects of betaine ingestion on HCTL in healthy subjects. We hypothesize that by increasing transmethylation capacity betaine supplementation reduces plasma Hcy and may thus decrease HCTL generation, resulting in improved insulin signaling and myofibril protein synthesis, and ultimately enhancing muscle and strength gains. Therefore, the purpose of this study was to investigate the sub-chronic effects of betaine on strength, power, and body composition during resistance training in experienced strength trained males. Additionally, urine HCTL was measured to determine if betaine affects performance by reducing plasma HCTL. We hypothesized that betaine supplementation would improve strength, vertical jump, limb CSA, and body composition between the 1^st^ week and 6^th^ week over placebo. We also hypothesized that betaine supplementation would reduce urinary HCTL over the course of 6 weeks.

## Methods

### Experimental design

To investigate the effects of betaine supplementation, subjects were matched, and randomly assigned to a treatment or placebo group in a double-blinded study. Subjects underwent 6 weeks of supplementation with either betaine or placebo administered in identical gelatin capsules. Before and after the treatment period skin fold and girth measurements were taken, and subjects completed a strength testing protocol. Additionally, urine was collected prior to treatment and at 2 week intervals thereafter.

### Subjects

Twenty three experienced recreationally strength trained males (weight: 86.8 ± 9.1 kg; training experience: 4.8 ± 2.3 months; BF%: 16.9 ± 8%) between the ages of 18 and 35 were recruited divided into two groups based on training experience (6 month intervals) and body fat percentage (2 percentage point intervals starting at 6%), and randomly assigned to receive either the treatment (*n* = 11) or placebo (*n* = 12). Medical histories were obtained to exclude medical, musculoskeletal, and endocrine disorders, concurrent nutritional supplementation, and anabolic drugs. Additionally, subjects must have met the inclusion criteria to be classified as experienced in resistance training [17]: previous consecutive resistance training equal to or greater than 24 months; a frequency of at least 3 resistance training sessions per week; at least 24 months experience in the back squat and bench press; and the ability to bench press a load equal to body weight and back squat at least 1.25 fold that of body weight. All subjects signed an informed consent form following verbal and written explanation of benefits and potential risks associated with participating in the study.

### Experimental controls

Subjects were required to complete a 3-day food diary, and were instructed to consume a similar quantity/quality of foods throughout the study in order avoid changes in nutritional status. Subjects were also required to perform all prescribed resistance training sessions, complete and submit training logs to the primary investigator on a weekly basis, and abstain from performing other structured exercise programs throughout the duration of the study. Subjects were required to render urine upon waking following an overnight fast. Limb girth, skin fold, strength, and power testing was carried out at the same time of day within 2 days prior to and immediately following the 6 week trial period. Prior to all exercise tests, subjects were familiarized with the assessment protocols. All methods and procedures were approved by the Institutional Review Board of Springfield College prior to data collection.

### Procedures

All testing was conducted at the Springfield College Human Performance Laboratory (HPL). Subjects were required to report to the HPL on two separate occasions (pre-treatment and post treatment) where height, nude body mass, skin fold, anthropometric measurements, and maximal strength testing was performed.

### Body fat

Lange skin fold calipers (Cambridge Scientific Industries Inc: Cambridge, MD) were calibrated before testing each subject, and measurements were performed by the primary investigator to eliminate inter-rater variability. Skin folds (mm) were measured on the right side of the body in the following rotation: sub-scapular (X_1_), abdominal (*X*_2_), triceps brachii (X_3_), and chest at the mix-auxiliary line (X_4_). Body density (BD) was estimated via the following equation [18]: BD = 1.03316 - .00164X_1_ + .0041H - .00144X_2_ - .00069X_3_ + .00062X_4_, and then used to estimate BF % [19]: BF % = [(4.57 / BD) – 4.142] × 100. Lean body mass (LBM) and fat mass (FM) were then calculated from the BF % and body weight.

### Cross sectional area

A 6-week trial period was chosen to allow for detectable changes in muscle CSA to occur. Changes in limb muscle mass have been demonstrated to be detectable via CSA measurements after four weeks of training and continue to increase week to week [20]. Limb muscle volume was assessed by evaluating differences in CSA via the Moritani and DeVries (MD) method [21]. The MD method is both sensitive (*SEE* = 3.25 cm^2^) and highly correlated (*r* = .98) to computed tomography, the gold standard of CSA measurement [22].

Girth and skin fold measurements were performed on the right limbs to determine CSA via the MD method. Cross sectional area of the arm was determined at the midpoint between the humeral greater tuberosity and lateral epicondyle, whereas CSA of the thigh was determined at the midpoint of the distance between the greater trochanter and lateral epicondyle of the femur. Skin fold measurements were performed three times at the four quadrants of the limb at the location where the circumference was measured. Cross sectional area was calculated via the following equation [21]: $\mathrm{CSA}=\pi \;\left[\frac{C}{2\pi }-\frac{\sum_{i=1}^{4}\mathrm{fi}}{4}\right]$, where *C* = limb circumference and $\overset{4}{\underset{i=1}{\sum }}\mathrm{fi}$ = sum of skin folds. All measurements were performed by the primary investigator to eliminate inter-rater variability. Distances from the proximal boney land mark (humeral greater tuberosity and greater trochanter) where measurements were performed were recorded and used again for post treatment measuring to minimize intra-rater variability.

### Strength and power testing

All strength and power testing was conducted under the supervision of a National Strength and Conditioning Association (NSCA) Certified Strength and Conditioning Specialist. Power was assessed via vertical jump using the Just Jump! Mat (Probotics Inc.: Huntsville, AL). Maximal strength was assessed with the free weight bench press and back squat. The heaviest resistance lifted in each exercise was considered the 1 RM. The bench press and back squat were chosen for strength assessment because: they are common exercises performed by weight lifters and the standardized strength training program in this study utilized the two exercises. Additionally, 1 RM testing has been shown to be a reliable (ICC = .96) [17] measure to assess changes in muscle strength following an exercise intervention.

All subjects completed a standardized dynamic warm up prior to performance testing. Following a 5-min rest subjects performed 3 trials of counter-movement vertical jumps separated by a 3-min rest. Vertical jumps were measured in inches on the Just Jump! mat. Subjects were instructed to perform a rapid lower body eccentric contraction followed immediately by a maximal intensity concentric contraction. Subjects were instructed to jump straight up and minimize any in-air hip flexion. The best of the three trials was recorded as vertical jump height.

Subjects were then given a 3-min rest prior to the strength specific warm ups. Subjects performed three sets of four repetitions with a progressively heavier load, three sets of one repetition with a progressively heavier load, and then a 3 min rest prior to attempting the first 1 RM. The first load used was 90% of the subject’s most recent 1 RM or predicted from the subject’s most recent RM [23]: 1-RM = 100 * rep wt / (101.3 – 2.67123 * reps). Loads were increased by 5 – 10% and 10 – 20% for bench press and squat, respectively, and then the 1 RM was determined in fewer than 5 sets with a rest interval of 3–5 min between sets. There were no significant differences in attempts between pre- and post-testing (3.4 ± .82, *p* = .71). The bench press 1 RM was tested first, and then a rest interval of at least 10 min was provided prior to determining the back squat 1 RM.

### Homocysteine thiolactone

HCTL is a toxic metabolite in humans and renal excretion serves as the primary method of HCTL elimination [14]. Urinary concentrations of HCTL are 100 fold greater than those found in the plasma [24]. Urine was rendered upon waking following an overnight fast prior to treatment administration (baseline) and at the end of week 2, 4 and 6 throughout the study. The urine samples were collected by the primary investigator on the same day that urine was rendered and stored in 1-mL aliquots at −80°C prior to being sent for analysis. Urine was analyzed for HCTL via the cation-exchange high pressure liquid chromatography (HPLC) at the Institute of Bioorganic Chemistry, Polish Academy of Sciences, Poznan, Dept. of Biochemistry and Biotechnology, Life Sciences University, Poznan, Poland, as described Jakubowski et al. [24-26]. The cation-exchange HPLC is highly sensitive with a 0.36 nmol/L detection limit [24].

### Treatments

Treatments were administered double blind and consisted of either a placebo (flour) or betaine (DuPont Nutrition & Health: Tarrytown, NY). The blind was not removed until all data had been collected. The primary investigator filled identical, unmarked gelatin capsules with either 0.42 g white flour or 0.42 g betaine. Subjects consumed three capsules (1.25 g) twice per day yielding an absolute total of 2.5 g betaine. This dosage was chosen because: betaine is safe at a dietary intake of 9 – 12 g/day [1]; 2.5 - 5 g betaine has been shown to significantly elevate plasma betaine [6]; 2.5 g positively affects strength performance [2,4]; and the average relative dosage (34.8 mg/kg-LBM) in the present study is similar to the average relative dosage (36.3 mg/kg-LBM) reported previously to improve performance [3]. Subjects were provided with 42 capsules per week and were required to log and submit consumption times in accordance with the treatment protocol.

### Training program

Subjects assigned to the betaine and placebo groups performed the same exercises, sets and repetitions during the six week investigation. A non-linear periodization (NLP) training program consisting of three 2-week micro-cycles was prescribed because NLP has been shown to produce larger increases in strength [27] and muscle volume [28] compared to linear or non-periodized programs. Upper body training was performed on Monday and Thursday, whereas lower body training took place on Tuesday and Friday. The prescription for exercises, training days, loads, rest period, warm ups, and resistance progression can be found in Table 1.

**Table 1:** Non-linear 6 week resistance training program

**Monday**	**Micro cycle 1**	**Micro cycle 2**	**Micro cycle 3**
Exercise	**Week 1 - 2**	**Week 3 - 4**	**Week 5 - 6**
Barbell Bench Press	3 × (12–15) / 90 sec	4 × (4–6) / 3 min	3 × (8–10) / 2 min
Barbell Incline Press	3 × (12–15) / 90 sec	4 × (4–6) / 3 min	3 × (8–10) / 2 min
Bent Over Barbell Row	3 × (12–15) / 90 sec	4 × (4–6) / 3 min	3 × (8–10) / 2 min
Seated Cable Row	3 × (12–15) / 90 sec	4 × (4–6) / 3 min	3 × (8–10) / 2 min
Biceps Barbell Curl	3 × (12–15) / 90 sec	4 × (4–6) / 3 min	3 × (8–10) / 2 min
Lying Triceps Extension	3 × (12–15) / 90 sec	4 × (4–6) / 3 min	3 × (8–10) / 2 min
**Tuesday**	**Micro cycle 1**	**Micro cycle 2**	**Micro cycle 3**
Exercise	**Week 1 - 2**	**Week 3 - 4**	**Week 5 - 6**
Free Weight Back Squat	3 × (8–10) / 2 min	3 × (12–15) / 90 sec	4 × (4–6) / 3 min
Barbell Rumanian Dead Lift	3 × (8–10) / 2 min	3 × (12–15) / 90 sec	4 × (4–6) / 3 min
Leg Extension Machine	3 × (8–10) / 2 min	3 × (12–15) / 90 sec	4 × (4–6) / 3 min
Abdominal Crunches	3 × (20–30) / 1 min	3 × (20–30) / 1 min	3 × (20–30) / 1 min
**Thursday**	**Micro cycle 1**	**Micro cycle 2**	**Micro cycle 3**
Exercise	**Week 1 - 2**	**Week 3 - 4**	**Week 5 - 6**
Barbell Military Press	3 × (12–15) / 90 sec	4 × (4–6) / 3 min	3 × (8–10) / 2 min
Wide Grip Front Lat Pull Down	3 × (12–15) / 90 sec	4 × (4–6) / 3 min	3 × (8–10) / 2 min
Dumbbell Row	3 × (12–15) / 90 sec	4 × (4–6) / 3 min	3 × (8–10) / 2 min
Dumbbell Lateral Shoulder Raise	3 × (12–15) / 90 sec	4 × (4–6) / 3 min	3 × (8–10) / 2 min
Alternating Curls with Dumbbells	3 × (12–15) / 90 sec	4 × (4–6) / 3 min	3 × (8–10) / 2 min
Triceps Extension with Cables	3 × (12–15) / 90 sec	4 × (4–6) / 3 min	3 × (8–10) / 2 min
**Friday**	**Micro cycle 1**	**Micro cycle 2**	**Micro cycle 3**
Exercise	**Week 1 - 2**	**Week 3 - 4**	**Week 5 - 6**
Standard Dead Lift	3 × (8–10) / 2 min	3 × (12–15) / 90 sec	4 × (4–6) / 3 min
Barbell Split Squat	3 × (8–10) / 2 min	3 × (12–15) / 90 sec	4 × (4–6) / 3 min
Prone Leg Curl Machine	3 × (8–10) / 2 min	3 × (12–15) / 90 sec	4 × (4–6) / 3 min
Hanging Leg Raises	3 × (20–30) / 1 min	3 × (20–30) / 1 min	3 × (20–30) / 1 min

Note: Volume is prescribed as: Sets × (Rep Range) / Rest Periods.

The training sessions were not monitored; however, subjects were required to submit training logs to the primary investigator on a biweekly basis (at the conclusion of each micro-cycle). Training volume was calculated as the sum of the load lifted multiplied by the number of repetitions performed during each week for the bench press and back squat, respectively. Work capacity for bench press and back squat was assessed by comparing percent improvement in training volume for each micro-cycle (week 1 vs. week 2; week 3 vs. week 4; week 5 vs. week 6).

### Statistical analysis

An independent samples *t*-test was used to examine differences between groups for pre-trial BF % and training experience. A 2 × 5 Mixed Factorial ANOVA with Repeated Measures was used to determine the difference between groups (placebo and betaine) and time for changes in urinary HCTL from baseline and week to week. Two 2 × 6 Mixed Factorial ANOVA with Repeated Measures were used to determine differences between groups and time for bench press and back squat work capacity at each training micro-cycle. If significant interactions were found, percent improvements at each micro-cycle was calculated and compared between groups with an independent samples *t*-test. Eight 2 × 2 Mixed Factorial ANOVAs with Repeated Measures were used to determine differences in arm CSA, thigh CSA, BF %, LBM, FM, vertical jump, bench press 1 RM, and back squat 1 RM between groups and time (pre- vs. post-trial). All statistical analyses were analyzed using Statistical Package for the Social Sciences (SPSS v. 19, IBM) and the alpha level was set at .05.

## Results

**Figure 1:**
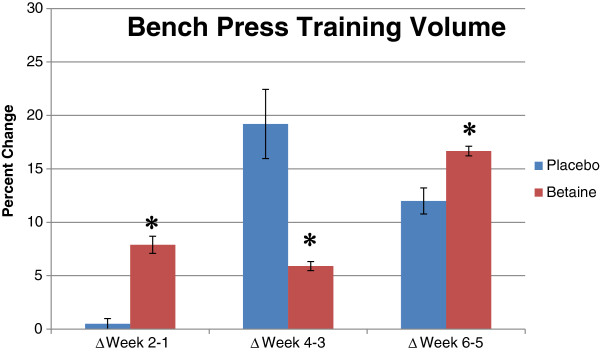
**Percent change in bench press volume for placebo (n = 12) and betaine (n = 11) for 3 training micro-cycles.** Note: * = Significantly (p < .05) different than placebo.

All values are presented as means ± standard deviations. A significant interaction (*p* = .001) between group and time existed for bench press work capacity (Figure 1). Bench press training volume increased with placebo at micro-cycles 2 and 3, and for betaine at micro-cycles 1 and 3 (Table 2). *Post hoc* analysis revealed the betaine group improved significantly more than placebo at micro-cycle one (7.89 ± 2.65% vs. 0.49 ± 1.69%, *p* = .001) and three (16.67 ± 1.51% vs. 12.00 ± 4.21%, *p* = .05); however, the percent improvement for placebo was significantly greater than betaine at micro-cycle two (19.2 ± 11.2% vs. 5.9 ± 1.4%, *p* = .001).

**Table 2:** Changes in bench press training volume (kg) for placebo (n = 12) and betaine (n = 11) between three micro-cycles

	**Pre**	**Post**	**∆**	***P***
**Micro Cycle 1**				
**Betaine**	2736 ± 463	2953 ± 500	216 ± 39	.01
**Placebo**	3154 ± 553	3170 ± 555	15 ± 70	.44
**Micro Cycle 2**				
**Betaine**	1755 ± 296	1858 ± 315	103 ± 25	.30
**Placebo**	2320 ± 406	2903 ± 672	583 ± 288	.01
**Micro Cycle 3**				
**Betaine**	2160 ± 365	2520 ± 427	360 ± 101	.01
**Placebo**	2481 ± 435	2779 ± 487	298 ± 62	.01

**Figure 2:**
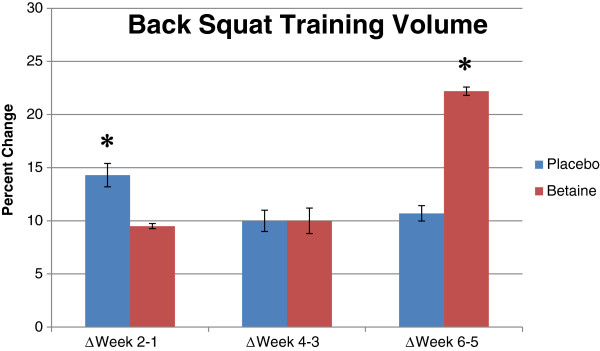
**Percent change in back squat volume for placebo (n = 12) and betaine (n = 11) for 3 training micro-cycles.** Note: * = Significantly (p < .05) different than placebo.

A significant main effect (*p* = .001) of time existed for squat work capacity. Both groups increased squat volume at the second week of each training micro-cycle (Figure 2 & Table 3); however, *post-hoc* analysis revealed the percent improvement in squat training volume was significantly greater with placebo than betaine at micro-cycle one (14.3 ± 3.8% vs. 9.5 ± 0.8%, *p* = .001), and significantly greater with betaine than placebo at micro-cycle three (22.2 ± 1.3% vs. 10.7 ± 2.5%, *p* = .001). There were no differences (*p* = .68) between groups for percent improvement at micro-cycle two.

**Table 3:** Changes in back squat training volume (kg) for placebo (n = 12) and betaine (n = 11) between three micro-cycles

	**Pre**	**Post**	**∆**	***P***
**Micro Cycle 1**				
**Betaine**	2760 ± 482	3022 ± 527	262 ± 43	.01
**Placebo**	3003 ± 695	3364 ± 779	360 ± 84	.01
**Micro Cycle 2**				
**Betaine**	3736 ± 652	4084 ± 712	347 ± 76	.01
**Placebo**	4015 ± 930	4444 ± 1030	428 ± 159	.01
**Micro Cycle 3**				
**Betaine**	2056 ± 357	2541 ± 444	484 ± 91	.01
**Placebo**	2350 ± 545	2655 ± 633	305 ± 85	.01

**Figure 3:**
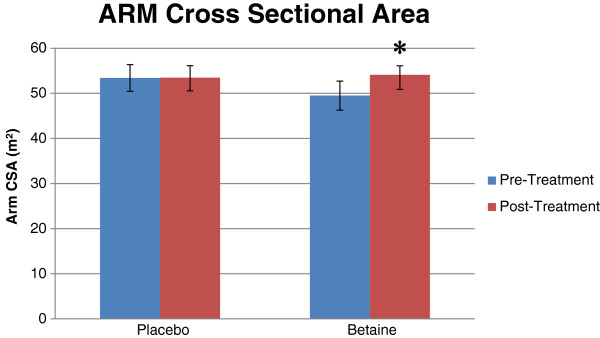
**Bar graph for arm cross sectional area (cm**^**2**^**) for placebo (n = 12) and betaine (n = 11) for pre- and post-treatment.** Note: * = Significantly (p < .05) different than pre-treatment.

No significant (*p* = .70) main effect or interaction existed between group and time for thigh CSA (Table 4). A significant (*p* = .03) interaction was found between groups and time for arm CSA (Figure 3). Arm CSA increased significantly post-trial vs. pre-trial with betaine but not placebo (Table 4).

**Table 4:** **Changes in thigh and arm cross sectional area (cm**^**3**^**) for placebo (n = 12) and betaine (n = 11) between pre- and post-treatment**

	**Pre**	**Post**	**∆**	***P***
**Thigh CSA**				
**Betaine**	85.0 ± 12.2	87.7 ± 12.2	2.7 ± 4.2	.254
**Placebo**	87.6 ± 17.7	89.0 ± 13.9	2.3 ± 10	.254
**Arm CSA**				
**Betaine**	49.5 ± 8.7	54.1 ± 6.6	4.6 ± 4.3	.01
**Placebo**	53.4 ± 10.2	53.5 ± 11.2	-.1 ± 5	.98

**Figure 4:**
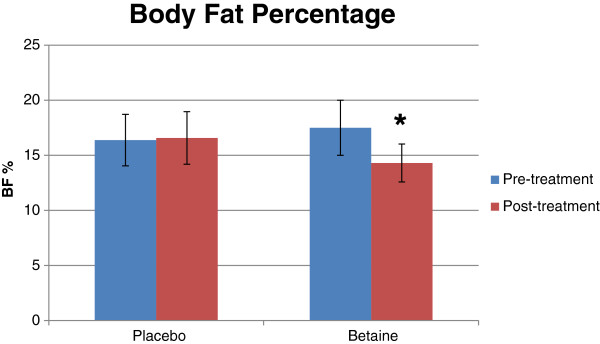
**Bar graph for body fat percentage for placebo (n = 12) and betaine (n = 11) for pre- and post-treatment.** Note: Significantly (p < .05) different than pre-treatment.

**Figure 5:**
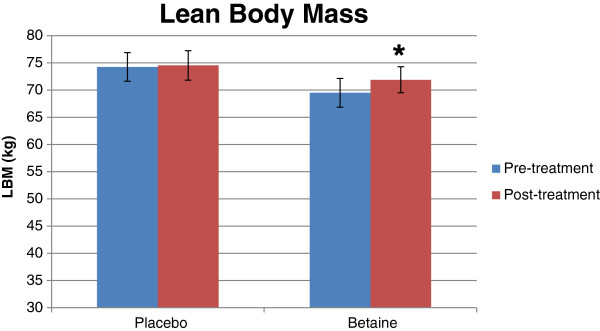
**Bar graph for lean body mass (kg) for placebo (n = 12) and betaine (n = 11) for pre- and post-treatment.** Note: Significantly (p < .05) different than pre-treatment.

All body composition data can be found in Table 5. Significant interactions between group and time were found for BF% (*p* = .007), LBM (*p* = .03), and FM (*p* = .01). BF% and FM both decreased significantly post-trial vs. pre-trial with betaine but not placebo (Figures 4, 5). Post-trial LBM increased significantly over pre-trial with betaine but not placebo.

**Table 5:** Changes in body composition for placebo (n = 12) and betaine (n = 11) for pre- and post-treatment

	**Pre**	**Post**	**∆**	***P***
**Body Fat (%)**				
**Betaine**	17.5 ± 8.3	14.3 ± 5.7	−3.2 ± 2.5	.01
**Placebo**	16.4 ± 8.1	16.6 ± 8.2	0.2 ± 2.7	.82
**Lean Body Mass (kg)**				
**Betaine**	69.5 ± 8.8	71.2 ± 7.9	2.4 ± 2.6	.01
**Placebo**	74.2 ± 9.1	74.5 ± 9.4	0.3 ± 2.6	.68
**Fat Mass (kg)**				
**Betaine**	15.0 ± 7.9	12.1 ± 5.4	−2.9 ± 2.0	.01
**Placebo**	14.8 ± 8.0	15.1 ± 8.5	0.3 ± 2.3	.68

Vertical jump, bench press 1RM and back squat 1Rm data can be found in Table 6. An interaction trend (*p* = .07) was found for vertical jump. Vertical jump decreased with placebo and increased in betaine. No significant (*p* = .99) interaction or main effect (*p* = .12) existed between group and time for bench press. A significant (*p* = .001) main effect for time was found for back squat 1 RM. Mean post-trial back squat 1 RM was significantly greater than pre-trial squat 1 RM; however, no significant interaction (*p* = .18) existed between group and time.

**Table 6:** Changes in vertical jump (cm), Back squat 1RM (kg), and Bench press 1RM (kg) for Placebo (n = 12) and Betaine (n = 11) for pre- and post-treatment

	**Pre**	**Post**	**∆**	***P***
**Vertical Jump**				
**Betaine**	68.1 ± 8.4	68.8 ± 8.4	0.8 ± 3.3	.45
**Placebo**	65.5 ± 10.4	63.0 ± 9.9	−2.5 ± 4.0	.09
**Bench Press**				
**Betaine**	118.2 ± 19.3	120.0 ± 20.3	1.8 ± 4.3	.20
**Placebo**	137.7 ± 25.0	140.0 ± 24.5	2.3 ± 6.0	.31
**Back Squat**				
**Betaine**	148.6 ± 26.7	151.4 ± 26.4	2.7 ± 4.5^*^	.09
**Placebo**	159.1 ± 38.8	164.5 ± 38.1	5.5 ± 4.0	.01

* Non Significant Time × Treatment Interaction: *p* = .18.

**Figure 6:**
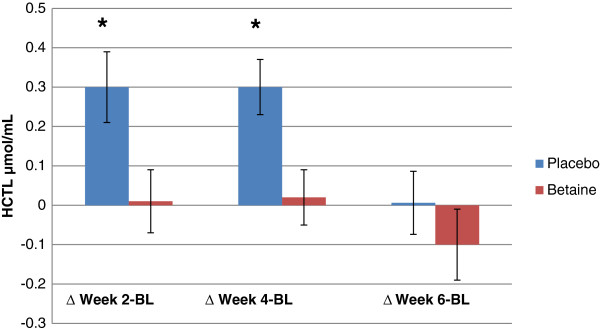
**Changes in urinary homocysteine thiolactone values for placebo (n = 12) and Betaine (n = 11) between baseline and three time intervals.** Note: * = Significantly (p < .05) different than betaine.

There was a trend (*p* = .06) for greater baseline HCTL concentrations in betaine. A significant (*p* = .002) interaction between group and time was found for urinary HCTL. The change in urinary HCTL with placebo was significantly greater than that of betaine between baseline and week 2, and baseline and week 4, respectively (Figure 6 & Table 7). No significant changes in HCTL were found for either group when comparing the change between week 2 and 4 or week 4 and week 6; however, a main effect of time was found when comparing week 6 to week 4.

**Table 7:** Changes in urinary homocysteine thiolactone (nmol/mL) for Placebo (n = 12) and Betaine (n = 11) between baseline and three time intervals

	**Concentration**	**∆ From baseline**	***P***
	**Baseline**		
**Betaine**	.037 ± .024^*^	NA	NA
**Placebo**	.019 ± .018	NA	NA
	**Week 2**		
**Betaine**	.038 ± .02	.001 ± .02	.95
**Placebo**	.049 ± .03	.029 ± .01	.01
	**Week 4**		
**Betaine**	.039 ± .01	.002 ± .01	.74
**Placebo**	.048 ± .02	.029 ± .01	.01
	**Week 6**		
**Betaine**	.027 ± .03	-.024 ± .03	.29
**Placebo**	.026 ± .02	.011 ± .03	.48

* Not significantly different than placebo at baseline: *p* = .06.

## Discussion

We hypothesized body composition would improve with 6 weeks of betaine supplementation. This hypothesis was supported by significant increases in lean mass, and decreases in fat mass and body fat percentage with betaine compared to placebo. Increases in arm CSA were found to be greater with betaine than placebo; however, thigh CSA did not increase in either group. We also expected strength and power performance to improve with betaine supplementation. While back squat 1 RM increased for both groups, there were no differences in improvement between betaine and placebo. There was a trend (*p* = .07) for greater vertical jump power with betaine versus placebo, however there were no increases in bench press 1 RM.

The improvements in lean mass, fat mass and body fat percentage with betaine supplementation contrast previous investigations [5,6]. Differences in methodology may explain these discrepancies: subjects in the previous studies were both sedentary and instructed not to exercise, whereas the subjects in the present study were currently training and given a structured exercise program. Betaine has been suggested to act as a nutrient partitioner and thereby accelerate lean mass gains in pigs. By increasing Hcy transmethylation, betaine spares Met, allows for more efficient use of dietary protein, and increases nitrogen retention [7]. Due to the inclusion of resistance training in this study but not previous studies [5,6], the demand for Met in the initiation of translation in protein synthesis was likely elevated, thereby leading to a greater utilization of elevated Met, and thus improvements in lean mass. Therefore, the results from the present study lend support to the hypothesis that the action of betaine to improve body composition in humans may be most effective when accompanied by exercise.

The increase in arm CSA in the betaine group compared to placebo was accompanied by an improvement in bench press work capacity. The greatest improvements in volume over placebo occurred during the first and third training micro-cycles, where subjects were instructed to perform 3 sets of 12–15 repetitions with 90 sec rest periods and 3 sets of 8–10 repetitions with 120 sec rest periods, respectively. Given the relationship between training volume and hypertrophy [29], betaine may have positively impacted muscle growth by promoting a greater training load over a series of subsequent workouts.

The improvements in bench press work capacity differ from previous studies where betaine did not improve single-set repetitions to fatigue at 75% [3] or 3 sets of repetitions to fatigue at 85% 1 RM [2]. In contrast, betaine improved work capacity for 10 sets of repetitions to fatigue at 50% 1 RM [4]. Given improved work capacity with higher volume resistance training prescriptions, and the lack of improvement during micro-cycle 2 which imposed less of a metabolic demand (4 sets of 4–6 repetitions with 3 min rest), it is likely that betaine poses the most ergogenic potential in resistance training exercise protocols that impose higher metabolic demands. Betaine is actively taken up by skeletal muscle during periods of stress, and may be ergogenic as an osmolyte by protecting sensitive metabolic pathways against cellular hypertonicity such as protein turnover, amino acid and ammonia metabolism, pH regulation, and gene expression [30]. Specifically, betaine maintains cellular hydration to protect myosin ATPase and myosin heavy chain proteins against denaturation by urea [31]. Moreover, the affinity of troponin for Ca^2+^_,_ and thus force production, is negatively affected by reductions in protein hydration [32].

Contrary to the changes in arm CSA, no differences in leg CSA were found between groups. Similar results have been reported in animal studies investigating the effects of betaine supplementation on carcass cuts where betaine supplementation improved shoulder and butt, but not ham meat yield [9]. Additionally, changes in upper body muscle thickness occur at a greater magnitude and earlier than do the lower extremities [33]. Therefore, it is possible that changes in thigh CSA may have occurred with a longer study period.

Although the back squat requires recruitment of the quadriceps femoris, it also has a high gluteal/hip requirement. Increases in muscle mass may have occurred predominantly in the gluteals as seen in animal studies, or the adaptations leading to greater back squat volume and 1 RM occurred separately from increased muscle CSA. Back squat work capacity increased for each group at each training micro-cycle; however, the betaine group improved nearly two-fold compared to placebo during micro-cycle three (4 sets of 4–6 repetitions with 3 min rest) which posed a higher neural and lower metabolic demand than the previous micro-cycles. These improvements in back squat work capacity contrasts previous results [34] whereby betaine did not improve mean or peak isokinetic power during 5 sets of 6 repetitions at 80% peak force. The improvements in work capacity at micro-cycle three but not micro-cycle one or two also contradict our hypothesis that betaine may be most ergogenic when combined with exercise protocols producing higher levels of metabolic stress. Given the improvement in bench press work capacity that also occurred at micro-cycle three but not two, and the lack of improvement with only 2 weeks of supplementation [2,4], it may also be that the effects of increased intramuscular betaine manifest over a longer period of time, and therefore require at least a 4–6 week ingestion period.

There were no differences between groups for back squat 1 RM improvements, and despite increases in bench press training volume with betaine, bench press 1 RM did not improve. This contrasts previous reports [2], and may be partially explained by difference in subject training status. Lee et al. employed recreationally trained subjects, whereas subjects in the present study averaged 4.8 years of training experience. The ability to make large performance gains, termed the “window of adaptation” [35], decreases with training experience. The “window of adaptation” was likely smaller for the subjects in the present study, thus reducing the ability to detect changes in strength. Finally, the primary aim of this study was to evaluate the effects of betaine on muscle growth; thus, the training program utilized was selected because it provided the greatest stimulation for hypertrophy. Given the high training status of the subjects, a strength concentrated program (i.e.: 4–6 sets of 1–3 repetitions) may have been needed to induce further improvements in bench press and back squat 1 RM with betaine supplementation.

There was a trend (*p* = .07) toward an increased vertical jump with betaine supplementation. The positive trend in the present study and improvements reported by Lee et al. [2] differs from the results reported by other researchers where vertical jump did not increase with betaine [3,4]. Variances in training prescription may account for these discrepancies. In Lee et al. and the present study subjects were assigned standardized training between testing sessions, whereas subjects in Hoffman et al. [4] and Trepanowski et al. [3] were not. Because detections in power improvements are compromised when power movements are not a regular part of training [34], future researchers should include exercises that train muscular contractile velocity when investigating the effects of betaine supplementation on power output.

We hypothesized that subjects would have high urinary HCTL values due to reduced Hcy transmethylational capacity; however, the results did not support this hypothesis. The normal range for urinary HCTL is .011-.473 nmol/mL [24]. Mean pretreatment HCTL was .028 nmol/mL (± .02 nnmol/mL), which suggests that the subjects began the study with low HCTL levels. Betaine supplementation attenuated the rise in HCTL observed in placebo at weeks 2 and 4, but did not appear to reduce HCTL values. Many subjects moved from the campus dormitories to live with their parents for the summer. It is possible that subjects had access to foods higher in protein quality and richer in fats and cholesterol than when living on campus, and this led to the increase in HCTL. Increases in dietary fat and cholesterol have been shown to increase plasma Hcy [36] as 3 Hcy are produced during the methylation of phosphatidylethanolamine in very low density lipoprotein synthesis. Thus, higher methionine and fat intakes may have increased Hcy generation, leading to higher levels of HCTL. Given the ability of betaine to increase Hcy transmethylation, it is possible that betaine supplementation attenuated the dietary induced rise in HCTL.

HCTL decreased in both groups between week 4 and week 6, although there was a trend for a reduction in HCTL when comparing week 6 to baseline with betaine and not placebo. While subjects were instructed to maintain the same diet throughout the study, many foods rich in betaine and folate come into season in June including spinach (0.3 mg/cup folate) and collard greens (0.2 mg/cup folate), and the consumption of two-three servings of folate rich food per day will reduce Hcy by 20% [37]. Because the start of June corresponded with week 4 of the study, it is possible that the consumption of local greens and the resultant increase in folate consumption may have reduced HCTL values in week 6.

Based on the small differences in HCTL changes, betaine supplementation may have impacted body composition via other mechanisms. Betaine has been shown to elevate plasma GH and IGF-1, and increase Akt phosphorylation in human skeletal muscle [38]. In mice betaine improves insulin sensitivity by restoring activation of IRS1 and the subsequent phosphorylation of PI3K/Akt by 50-100% in a concentration-dependent manner [39]. Thus, it is possible that by elevating anabolic hormones and enhancing downstream cellular signaling, betaine may have improved muscle protein synthesis, thus leading to an increase in lean mass. Finally, because betaine is a powerful osomylte, the increases in lean mass may have been due to cellular swelling without an appreciable increase in myofibril protein accretion.

### Limitations

The MD method for estimating muscle CSA presents a potential limitation when interpreting the limb CSA results of the present study. The *SEE* for the MD method is 3.25 cm^2^. In the present study, the betaine group increased arm CSA by 4.6 cm^2^ compared to a 0.1 cm^2^ decrease with placebo. The difference in change for thigh CSA between betaine and placebo was 2.7 and 1.4 cm^2^, respectively. It is possible that a non-significant difference in arm CSA change or a significant difference in thigh CSA change may have been observed if CSA was measured differently. Future studies examining the effects of betaine on muscle CSA change should utilize an analysis with a lower *SEE*.

Caution should also be taken when interpreting the HCTL results. The primary aim in the present study was to determine the effectiveness of betaine supplementation to improve strength and body composition in weight trained males. A secondary aim was investigate if a relationship between changes in HCTL values and body composition or performance existed. Because improvements in strength were reported in previous studies without controlling for micronutrients [2,4], subjects were instructed to consume a similar quantity and quality of foods throughout the study to control for energy and protein intake. Because subject diets were not analyzed for micronutrients, it is possible that dietary fluctuations in folate, betaine, or other B-vitamin consumption occurred and influenced urinary HCTL. Future studies should provide standard control meals and/or analyze micronutrient intake to investigate clinical relationships between betaine supplementation and HCTL.

## Conclusions

In summary, the major findings of the present study are that 6 weeks of betaine supplementation improved body composition, muscle size, work capacity, attenuated a rise in HCTL, tended to improve power, but not strength in resistance trained men. Further work is warranted to confirm any role of HCTL on body composition compared to other mechanisms like lipogenic enzymatic activity, growth hormones, cellular signaling, or gene expression. Betaine attenuated an increase in urinary HCTL; however, because strength trained men in this study had low baseline HCTL betaine likely affected body composition via another mechanism. HCTL has been implicated in vascular disease [40], insulin resistance [13], diabetic retinopathy [41], seizures, and Alzheimer’s disease [42]. Thus, future investigations are needed to evaluate the clinical ability of betaine to reduce HCTL in at risk populations with elevated Hcy.

## Competing interests

DuPont Nutrition & Health (Tarrytown, NY) provided funding for this project. SASC is employed by DuPont Nutrition & Health. All other authors declare they have no competing interests. All authors involved collected, analyzed, or interpreted results from this study. Publication of these findings should not be viewed as endorsement by the editorial board of the Journal of International Society of Sports Nutrition.

## Authors’ contributions

JMC was the primary investigator, designed the study, obtained grant funds, supervised subject recruitment, data acquisition, data specimen collection, and manuscript preparation. MWR, RG, and HJ performed data specimen analysis. JMC was primarily responsible for writing the manuscript. TM, RW, SASC, and VP made substantial contributions to manuscript writing and preparation. All authors read and approved the final manuscript.
